# Does Cognitive Load Influence Moral Judgments? The Role of Action–Omission and Collective Interests

**DOI:** 10.3390/bs15030361

**Published:** 2025-03-13

**Authors:** Mufan Zheng, Liying Wang, Yueying Tian

**Affiliations:** Department of Psychology, Wuhan University, Wuhan 430072, China; 18956240891@163.com (L.W.); 2022301132015@whu.edu.cn (Y.T.)

**Keywords:** moral judgments, cognitive load, hybrid dual-process model, action propensity, collective versus individual interests

## Abstract

This study aimed to investigate the impact of cognitive load on moral judgments while incorporating action propensities and collective interests as variables. Study 1 (*N* = 102) used the dot matrix memory task to manipulate cognitive load, and participants made moral choices in action dilemmas and omission dilemmas. The findings revealed that when confronted with action moral dilemmas, participants in the high-cognitive load group exhibited a greater inclination towards utilitarian responses compared to those in the low-load group. However, cognitive load did not affect utilitarian choices in omission moral dilemmas. Study 2 (*N* = 100) further introduced the identities of protagonists in dilemmas involving conflicts between collective and individual interests. When facing a collective–individual interest conflict, participants under high cognitive load were more inclined to prioritize collective interests over individual interests compared to those under low load. Additionally, participants were more likely to choose collective interests in omission moral dilemmas than in action dilemmas. The impact of cognitive load on moral judgments was also influenced by the identities of the protagonists.

## 1. Introduction

In daily life, people often make choices involving ethical issues. Moral judgments are defined as evaluations (good vs. bad) of the actions or characters of a person that are made with respect to a set of virtues held to be obligatory by a culture or subculture (Haidt, 2001). The study of moral judgments typically employs the moral dilemma paradigm, which distinguishes two moral tendencies: deontology and utilitarianism (e.g., J. Greene et al., 2008). Utilitarianism promotes ‘the greatest good for the greatest number’; People who support utilitarianism concern maximizing benefits, the good, or utility (Bentham, 1789/1948; Darwall, 2023a; Kant, 1959). In contrast, deontology follows what is right and takes a certain universal value by checking whether certain qualities of actions against rules must be honored, thereby setting up constraints on actions. It concerns duty and responsibility, what must be done and what must not be done—regardless of the benefits (Darwall, 2023b).

Research on cognitive load’s impact on moral decisions has yielded conflicting results. Some studies suggest that cognitive load reduces utilitarian tendencies (Conway & Gawronski, 2013; Trémolière & Bonnefon, 2014; Trémolière et al., 2012), while others found no such effect (Bialek & De Neys, 2017; Gawronski et al., 2017; Tinghög et al., 2016).

Most studies use sacrificial moral dilemmas to explore moral reasoning, locating individuals’ choices on a continuum between utilitarianism and deontology (J. Greene et al., 2008; Tinghög et al., 2016). However, this methodology often neglects the role of action tendencies. Responses to sacrificial dilemmas might be shaped by both moral norms and action preferences (Crone & Laham, 2017; Cushman & Greene, 2012). Recently, Gawronski et al. (2017) discovered that cognitive load impacts inaction tendencies but not consequence tendencies, underscoring the significance of action tendencies.

Despite this, our primary interest lies in understanding how cognitive load affects individuals’ ultimate decisions in moral dilemmas. Therefore, our study included consideration of action tendencies, comparing how cognitive load influences individuals’ final moral decisions in scenarios where action is demanded versus those where it is not.

### 1.1. Moral Judgments

Traditionally, researchers used Greene’s dual-processing model (J. Greene & Haidt, 2002; J. Greene et al., 2004, 2008; J. Greene, 2015) to explain the impact of cognitive load on moral reasoning. According to this model, deontological judgments are typically driven by an immediate intuitive response to moral dilemmas, whereas utilitarian judgments necessitate more effortful and deliberative cognitive processes. According to this model, cognitive load depletes cognitive resources, resulting in fewer utilitarian decisions. However, this assumption is only partially supported by empirical evidence (e.g., Spears et al., 2020; Li et al., 2018).

The dual-processing model of moral judgments has faced challenges due to conflicting findings on utilitarian and deontological decisions. While some studies show longer reaction times for utilitarian choices and increased deontological decisions under time pressure (J. Greene et al., 2008; Suter & Hertwig, 2011), others found no difference in response times or even increased utilitarian tendencies under pressure (Baron & Gurcay, 2016; Gurcay & Baron, 2016; Tinghög et al., 2016). This suggests that the dual-processing model may not account for all factors influencing moral judgments.

To address these gaps, the hybrid dual-process model (Bago & De Neys, 2019a, 2019b) proposes that initial responses to moral dilemmas can be both deontological and utilitarian, with the relative activation strength of these intuitions determining whether the initial response is revised during reflection. The stronger the activation, the more likely the intuition will guide the initial response. Conversely, the larger the difference in activation strength between the two intuitions, the less likely the initial response will be modified during subsequent reflection. Under cognitive load, individuals’ cognitive resources are depleted and they rely more on intuition, leading to either deontological or utilitarian decisions, which explains the inconsistent impact of cognitive load on moral judgments.

Additionally, the conflict model (Gurcay & Baron, 2016) suggests that moral judgments depend on the interplay between dilemma difficulty and personal competence. When these factors align, moral judgments become more complex, while greater discrepancies between them lead to more definitive utilitarian or deontological choices.

### 1.2. Action Propensity

The moral dilemma paradigm is commonly used in moral judgment research, with examples such as the trolley and footbridge dilemmas (Foot, 1967; Thomson, 1976). These scenarios typically involve deciding whether to sacrifice a few to save many, assessing the moral acceptability of such actions.

Crone and Laham (2017) noted that standard sacrificial dilemmas conflate preferences for utilitarianism versus deontology with action tendencies (action vs. inaction). In classical sacrificial dilemmas, utilitarianism aligns with action, requiring participants to actively intervene to make utilitarian choices. However, people are inherently sensitive to action, and they think that directly causing harm is worse than passively allowing it. This is known as the action–omission effect (Ayer, 1936; Bostyn et al., 2021; DeScioli et al., 2011). Cushman and Greene (2012) argue that actions are perceived as more causally responsible and blameworthy than inactions, influencing ethical judgments (Bohner et al., 1988; Henne et al., 2019).

It is necessary to clarify the definitions of action dilemma and omission dilemma. The definitions used in this study may differ from the philosophical distinction and the common everyday understanding, which typically views action as actively harming others and omission as failing to help someone in danger. To avoid confounding factors, the outcomes of action in action dilemmas and omission in omission dilemmas were set to be the same, meaning they were always associated with the outcomes of utilitarian choices. Action is defined as actively harming others to achieve a greater good, i.e., taking action to realize a utilitarian moral choice. In contrast, omission is defined as not acting in a scenario to achieve and implement the utilitarian moral theory. This distinction has also been employed by many previous psychological researchers (e.g., Bostyn & Roets, 2016; Gawronski et al., 2017).

When making ethical decisions, individuals consider factors such as causal responsibility and intention (Cushman et al., 2013; Malle et al., 2014; Trémolière & Djeriouat, 2016). Rosas et al. (2019) suggest that inaction can also be a utilitarian factor, with scenarios requiring no action to achieve the greater good activating stronger utilitarian intuitions than those requiring active intervention.

In this study, we adapted four classical sacrificial dilemmas from Bialek and De Neys (2017) into omission dilemmas, where participants decided whether to prevent another person from acting. In action dilemmas, people need to choose whether to take action to make a utilitarian choice, and in omission dilemmas, people decide whether not to act to make a utilitarian choice. This design distinguishes between action tendencies and adherence to moral theories. The key difference lies in the perceived responsibility for outcomes (Bohner et al., 1988; Henne et al., 2019). According to the conflict model, adjusting the number of utilitarian factors in dilemmas can influence the utilitarian pull (Rosas et al., 2019). Therefore, we can infer that omission dilemmas have a stronger utilitarian pull due to reduced responsibility for harm, leading to stronger utilitarian intuitions compared to action dilemmas, where utilitarian and deontological intuitions are more balanced (a 66.3% average rate of making a sacrifice in action dilemmas) (Bialek & De Neys, 2017). Thus, participants in omission dilemmas are more likely to make utilitarian decisions and less likely to change them during deliberation due to experiencing a greater difference in the strength of utilitarian and deontological intuitions.

### 1.3. Cognitive Load and Moral Judgments

Cognitive load refers to the amount of mental effort required by an individual’s working memory (Sweller, 2017). The influence of cognitive load on moral judgments, particularly in sacrificial dilemmas, is a subject of ongoing debate. Some research indicates that an increased cognitive load may diminish the propensity for utilitarian decisions. For example, Trémolière and Bonnefon (2014) and Trémolière et al. (2012) manipulated cognitive load using a dot memory task and observed a subsequent decrease in utilitarian choices. Similarly, Conway and Gawronski (2013) employed the process dissociation technique to separate deontological and utilitarian tendencies in moral thinking. They found that the act of memorizing a string of digits resulted in a reduction in utilitarian parameters. Additionally, traditional analyses showed that cognitive load led to fewer utilitarian choices in moral dilemmas.

This may be because utilitarian decisions require more cognitive resources, and tasks that impose these resources can hinder System 2, which is responsible for analytical thinking. According to J. D. Greene’s (2009) dual-process model, if utilitarian judgments are modifications of intuitive deontological judgments (System 1), then under cognitive load, individuals may be less likely to consider utilitarian options, reducing the likelihood of making utilitarian decisions.

J. Greene et al. (2008) provided further support for this theory. They used a digit search task to examine the effects of cognitive load on utilitarian decision-making. Although cognitive load did not significantly affect moral judgments, it increased the reaction time for utilitarian judgments by approximately 0.75 s, without significantly affecting the reaction time for deontological judgments. This suggests that utilitarian decisions require more analytical thinking, while deontological decisions are more impulsive, supporting the dual-process model.

However, other research presents conflicting findings. Tinghög et al. (2016) used an attention control video as a cognitive load task and found that cognitive load only affected moral judgments in the fumes dilemma, not in other classical dilemmas. Bialek and De Neys (2017) found that while high cognitive load reduced utilitarian choices in conflict dilemmas, it did not affect sensitivity to conflicting moral views. Gawronski et al. (2017), based on the CNI model, found that cognitive load does not affect consequentialist tendencies but impacts inaction tendencies. These studies challenge Greene’s dual-process model, suggesting it may not fully explain the impact of cognitive load on moral judgments.

Therefore, we aimed to explore the influence of cognitive load on moral judgments by considering action tendencies and further investigated which theoretical model better reflects its actual impact on individual moral judgments.

Bialek and De Neys (2017) introduced the hybrid model, suggesting that utilitarian thinking can arise from System 1, alongside deontological intuitions. They used a dot matrix task to deplete cognitive resources and found that cognitive load did not affect moral judgments or decision confidence in classic dilemmas, including the trolley, plane, cave, and hospital dilemmas. This supports the hybrid model, indicating that utilitarian and deontological intuitions can coexist. However, action tendency was not considered in Bialek and De Neys (2017), and the present study aimed to take this factor into consideration.

Research shows that actions are perceived as more causally responsible for negative outcomes than omissions (Feldman, 2019; Feldman & Albarracín, 2017). Negative stimuli often trigger more deliberation than positive ones (Bostyn & Roets, 2016). The action–omission effect prompts participants to consider their causal responsibility when they hesitate whether to act to harm others. This element can be considered as a negative stimulus and elicits deliberation. Bostyn and Roets (2016) posit that this effect activates individuals’ causal evaluative cognitive processes, leading them to be less inclined to engage in harmful actions and make fewer utilitarian judgments. In action dilemmas, participants consider their causal responsibility, which requires cognitive resources and leads to fewer utilitarian judgments. Conversely, in omission dilemmas, where no action is needed, the utilitarian pull is stronger, and judgments are less affected by cognitive load. Therefore, we hypothesize that under low cognitive load, individuals will make fewer utilitarian judgments in action scenarios compared to omission scenarios. However, this difference will disappear under high cognitive load, as deliberation is disrupted.

### 1.4. Conflicts Between Individual and Collective Interests

Self-interest is a fundamental human tendency that often conflicts with the inclination to prioritize collective interests, as noted by Hobbes (1651) in Leviathan. Numerous studies in ethics explore the interplay between individual and collective interests. In Eastern collectivist cultures, moral decisions tend to favor the collective. For example, children in collectivist cultures are more likely to break rules to help the group (Fu et al., 2007), and Easterners generally adhere to norms of respect for authority and loyalty more than Westerners (Graham et al., 2011). The classic moral dilemma of sacrificing a few to save many provides a framework for examining this conflict. Researchers have also incorporated personal and familial interests into moral dilemmas to better understand decision-making in such conflicts (Bago & De Neys, 2019b; Hao et al., 2015; Lammers & Stapel, 2009).

In this study, we aimed to explore the conflict between individual and collective interests to further validate the hybrid process model. We adapted sacrificial dilemmas to emphasize this conflict, with protagonists in collective roles (e.g., railroad employees and military commanders) facing the choice of sacrificing family members for the greater good. This adaptation reframes the utilitarian decision as a conscious sacrifice of individual interests for collective benefits. We also used action and inaction dilemmas to examine the impact of cognitive load on decisions involving individual and collective interests.

Bago et al. (2021) found that conflicts between altruistic and selfish inclinations activate both types of intuitions, influencing moral judgments. According to the hybrid model, intuitions with higher activation levels are more likely to shape initial decisions, with subsequent adjustments depending on the relative strength of these intuitions (Bago & De Neys, 2019b). In dilemmas involving collective vs. individual interests, cognitive engagement is needed to resolve the conflict, making decisions more susceptible to cognitive load (Bago et al., 2021).

In Study 2, we adapted dilemmas from Bago and De Neys (2019b) involving sacrificing family members for the greater good, where the acceptance rate of sacrifice is close to 50% and participants are more likely to change their initial choices compared to other types of dilemmas. This suggests a minimal difference in activation intensity between collectivist and self-interested intuitions, making moral judgments more sensitive to cognitive load. In Chinese social norms, collective-interest decisions are often praised, diminishing the action–omission effect in blame judgments.

Existing research shows that positive events do not automatically trigger causal attributions, so judgments that elicit praise do not evoke the action–omission effect (Bostyn & Roets, 2016). This means that the utilitarian pull linked to inaction disappears and that the activation intensity of collectivist and self-interested intuitions remains consistent across action and omission dilemmas. Therefore, we hypothesize that cognitive load will influence collective-interest judgments in both action and omission dilemmas, with different collective identities introducing additional influences. In East Asia, influenced by Confucianism, Daoism, and Buddhism, individuals in leadership roles may exhibit stronger collectivist inclinations (Chou et al., 2024), so we expected that individuals identifying as soldiers or exploration team leaders may exhibit stronger collectivist inclinations and weaker self-interested motivations, leading them to rely more on collective intuitions under cognitive load.

## 2. Study 1

Bago and De Neys (2017, 2019a, 2019b) provided evidence for the existence of utilitarian intuitions and subsequently proposed a hybrid model. The primary objective of Study 1 was to scrutinize the hybrid model by investigating the influence of cognitive load and action tendency (a factor of utilitarianism) on individuals’ moral judgments.

If individuals’ moral reasoning processes are consistent with the hybrid model, then cognitive load will not impact utilitarian judgments in omission moral dilemmas. Conversely, in action moral dilemmas, the presence of cognitive load will predispose individuals towards making utilitarian decisions. However, if dual-processing model (J. Greene & Haidt, 2002; J. Greene et al., 2004, 2008; J. Greene, 2015) holds true, then it is expected that cognitive load will exhibit a negative correlation with the probability of making utilitarian decisions in both action and omission moral scenarios.

### 2.1. Method

The experiment was conducted in a 2 (cognitive load: high/low) × 2 (action tendency: action/omission) mixed design, where cognitive load was the between-subjects variable, action tendency was the within-subjects variable, and the dependent variables were utilitarian decision-making.

#### 2.1.1. Participants

A total of 102 university students were randomly assigned to the high- and low-load groups. Among them, 46 (45.10%) were male and 56 (54.90%) were female; the mean age of the participants was 21.88 years (*SD* = 2.90, 18–39 years). All participants were physically and mentally healthy and participated in the experiment voluntarily. At the end of the experiment, the participants were given a certain amount of compensation as a reward for participating in the experiment.

#### 2.1.2. Materials and Procedures

This experiment adopted four types of moral dilemmas in Bialek and De Neys’ (2017) study: the trolley, airplane, hospital, and cave dilemmas. All dilemmas had the same core structure, where the participants had to decide whether to sacrifice the life of the few to save the majority (the ratio was 1:5). All four moral dilemmas were written in two versions, i.e., (i) action moral dilemmas, in which the participants needed to decide whether to initiate the act of sacrifice; and (ii) omission moral dilemma, in which the participants needed to decide whether to prevent another person from performing the act of sacrifice. Each participant completed a total of eight moral decisions, and the order of the eight moral dilemmas was randomized.

Each moral dilemma was presented in two parts. First, the general background information was presented (shown in italics in the example), and the participants read it and then pressed the space bar to go to the next page. The second part was then presented and participants were asked about their personal willingness to act (“Would you do X?”). The participants entered their answer by pressing the corresponding key on the keyboard. When the second part was presented, the textual material from the first part was presented again at the same time. A moral dilemma (the trolley dilemma) from the experimental material is presented below as an example:

A trolley traveling on a track is out of control, and there are five workers repairing the track just ahead of the track. The trolley is traveling down the track towards the five workers. If the trolley continues, the five workers will be killed.

**Action Moral Dilemma:** You are standing not far from the track and there is a joystick next to you. If you press the joystick, the trolley will be diverted to another turnoff. Unfortunately, you notice that there is also a worker on the turnoff at this time. If the turnoff is switched, this man will be killed, while the other five will be saved. Would you press the joystick to turn the train?

**Omission Moral Dilemma:** You are standing not far from the track and there is a joystick next to you. If you press the joystick, the trolley will be diverted to another turnoff. Unfortunately, you notice that there is also a worker on the turnoff at this time. If the turnoff is switched, this man will be killed, while the other five will be saved. At this point your friend walks up to the joystick and intends to press it. You are stronger than your friend and you can stop him from pressing the joystick. Would you stop your friend from pressing the joystick?

**Dot Matrix Memorization Load Task**: To manipulate the participants’ cognitive load in the experiment, the participants were asked to complete a dot matrix memorization task concurrently with their moral decision-making (Miyake et al., 2001; Trémolière et al., 2012). After the participants read the first part of the dilemma, the dot matrix was presented. Next, the participants read the second part of the dilemma and made a decision. After the participants made a decision, they were presented with four matrices with different dot patterns and they had to choose the matrix they were asked to memorize. Finally, the participants were given feedback on whether they memorized the correct matrix.

**Figure 1:**
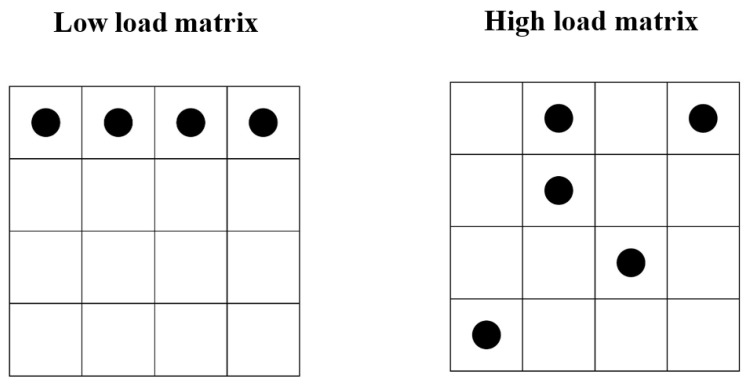
Example of high and low cognitive load matrices.

Half of the participants were randomly assigned to the high-load group and the other half to the low-load group. In the high-load condition, the matrix consisted of five dots arranged in a complex 4 × 4 network distribution pattern, and previous research has demonstrated that this demanding load task effectively interferes with cognitive processing systems during decision-making (Miyake et al., 2001; De Neys, 2006; Johnson et al., 2016). The four matrix options were all 4 × 4 matrices consisting of five dots, where one incorrect option had three dots in the same position as the correct option and two incorrect options had one dot in the same position as the correct option. In the low-load condition, the matrix consisted of four points in the same row or column. Examples of matrices in the high- and low-cognitive load conditions are shown in Figure 1.

The presentation time of the matrix to be memorized in the low-load condition was 2000 ms. To ensure that the participants could recognize complex matrices in the high-load condition, the presentation time of the matrix in the high-load condition was increased to 3000 (Trémolière et al., 2012).

### 2.2. Results

#### 2.2.1. Dot Matrix Memorization Load Task

The dot matrix memorization load task resulted in an average accuracy rate of 89.67% (*M* = 14.35, *SD* = 1.42). In the low-load condition, the mean correctness reached 88.37% (*M* = 14.14, *SD* = 1.57). In the high-load condition, the mean correctness reached 90.93% (*M* = 14.55, *SD* = 1.24). This indicates that most of the participants completed the cognitive load task seriously. However, there were still participants with low correct memorization rates, which implies that participants may have neglected the cognitive load task if they did not memorize the matrix correctly. To avoid this potential effect, data from participants with a correctness rate of 75% or less (*n* = 5, 4.90% of the total) were excluded from this study (Bialek & De Neys, 2017). The final data consisted of 97 participants (53 females, 44 males; mean age: 21.95, *SD* = 2.95), with a mean correctness of 89.71% (*M* = 14.35, *SD* = 1.16) for memorization in the low-load condition and 91.96% (*M* = 14.71, *SD* = 0.94) in the high-load condition.

#### 2.2.2. Moral Judgments

In each moral context, the participants were required to decide whether to sacrifice one to save more lives. The participants’ moral decisions were coded, and the mean willingness of participants to make utilitarian decisions in each dilemma was calculated. In the action moral dilemma, “would” was a utilitarian decision and “would not” was a deontological decision. In the omission moral dilemmas, because the participants needed to decide whether to stop the other person who was committing the sacrificial act, “would not” was a utilitarian decision and “would” was a deontological decision. Participants’ utilitarian choices were coded as “1”, and deontological choices were coded as “0”.

A 2 (cognitive load: high/low) × 2 (action propensity: action/omission) two-way repeated-measures ANOVA was conducted on moral judgments. The ANOVA results showed a non-significant main effect of cognitive load: *F*(1, 95) = 0.82, *p* = 0.368, *η_p_*^2^ = 0.01. The main effect of action propensity was significant: *F*(1, 95) = 4.90, *p* = 0.029, *η_p_*^2^ = 0.05. The participants were more likely to make utilitarian judgments in situations where inaction would lead to a utilitarian decision (*M* = 0.73, *SD* = 0.33) compared to action moral dilemmas (*M* = 0.64, *SD* = 0.34). The interaction between action preference and cognitive load was significant: *F*(1, 95) = 6.17, *p* = 0.015, *η_p_*^2^ = 0.06. Based on the effect size of the interaction effect, the statistical power was calculated using G*Power’s post hoc test. The results showed that 1 − *β* = 0.99.

Using syntax in SPSS 26 to perform a simple test (the LSD test), the results showed that there was no difference between the participants in the high-load group (*M* = 0.70, *SD* = 0.37) and the low-load group (*M* = 0.75, *SD* = 0.28) when they made moral judgments in omission moral dilemmas (*p* = 0.495), but in action moral dilemmas, the high-cognitive load group made more utilitarian decisions (M = 0.71, SD = 0.36) compared to those in the low-cognitive load group (*M* = 0.57, *SD* = 0.31; *p* = 0.035). Participants in the low-cognitive load group made more utilitarian decisions when they did not need to act (*M* = 0.75, *SD* = 0.28) compared to actively engaging in hurtful behaviors (*M* = 0.57, *SD* = 0.31; *p* = 0.001), whereas participants in the high-cognitive load group showed no difference in their moral judgments in the action moral dilemmas (*M* = 0.71, *SD* = 0.36) and omission moral dilemmas (*M* = 0.70, *SD* = 0.37; *p* = 0.85).

### 2.3. Discussion

Study 1 examined the influences of cognitive load and action–omission moral dilemmas on utilitarian judgments using a dot matrix memory cognitive load task. In the context of action moral dilemmas, participants subjected to a high cognitive load demonstrated a greater propensity to make utilitarian moral decisions compared to their counterparts in a low-cognitive load condition. However, no significant difference was observed in the utilitarian judgments of the two groups when faced with omission moral dilemmas.

In action dilemmas, participants in the high-cognitive load condition exhibited an increased tendency towards utilitarian choices compared to those in the low-cognitive load condition, which is consistent with our hypothesis. The action–omission effect in blame judgments automatically stimulates participants’ contemplation of causal responsibility attribution (Bostyn & Roets, 2016). When cognitive resources are sufficient, participants are more likely to reduce utilitarian choices due to considerations of their responsibility for harmful actions. However, with high cognitive load, individuals’ deliberation about the causal responsibility for actions is interfered with, leading them to make more utilitarian decisions. In omission dilemmas, since participants are not troubled by the need to take responsibility for causing harm, they are not affected by cognitive load.

The findings indicate that in moral dilemmas with varying degrees of activation of utilitarian and deontological intuitions, participants’ utilitarian inclinations are influenced by cognitive load in diverse ways. Moreover, cognitive load is less likely to disrupt participants’ judgments when the intensity of activation of utilitarian and deontological intuitions differs significantly. This finding aligns with our initial hypothesis and is consistent with the hybrid model (Bago & De Neys, 2019a).

## 3. Study 2

The study of utilitarian and deontological judgments, along with their influencing factors, has been a central focus within the realm of moral judgment. However, other facets of moral judgment warrant further exploration, particularly the tension between collective and individual interests.

Study 1 verified the rationality of the hybrid dual-process model in explaining utilitarian–deontological judgments. Nevertheless, other factors can also influence the degree of utilitarian inclination in moral contexts, such as collective interests. When people face a dilemma with a conflict between collective and individual interests, the emphasis on a collectivist identity introduces new utilitarian considerations. For instance, the perception of the military as a paragon of righteousness is deeply ingrained in societal consciousness. Consequently, participants’ collectivist instincts may be more robustly triggered when they assume the role of a military base commander or an exploration team leader in moral dilemmas.

If an individual’s moral reasoning aligns with the hybrid model, then there should be no discernible difference in the choices between collective and individual interests among participants in the high-load and low-load groups when the protagonist’s identity is that of a military figure or an exploration team leader. However, in moral dilemmas involving different identities, cognitive load can impact an individual’s decision-making process in favor of the collective good.

By contrast, according to Greene’s dual-process model (J. Greene & Haidt, 2002; J. Greene et al., 2004, 2008; J. Greene, 2015), participants’ decisions regarding collective interests should be equally influenced by cognitive load, irrespective of their assumed identity in moral dilemmas.

### 3.1. Method

The experiment was conducted in a 2 (cognitive load: high/low) × 2 (action propensity: action/omission) × 4 (identities: same railroad company employee/military base commander/same hospital security guard/expedition team chief) mixed design, where cognitive load was the between-subjects variable and action propensity and identities were the within-subjects variables. The dependent variables were collective-interest judgments.

#### 3.1.1. Participants

One hundred current university students were randomly assigned to high- and low-load groups. Among them, 23 (23%) were male and 77 (77%) were female; the mean age of the participants was 22.05 years (*SD* = 2.66, 18–29 years). All participants were physically and mentally healthy and participated in the experiment voluntarily. At the end of the experiment, the participants were given a certain amount of compensation as a reward for participating in the experiment.

#### 3.1.2. Materials and Procedures

Study 2 draws on Bialek and De Neys’ (2017) study on the effect of cognitive load on moral judgments using four classical moral dilemmas: the trolley, airplane, hospital, and cave dilemmas. All dilemma texts had the same core structure, where participants had to decide whether to sacrifice the life of the few to save the majority (the ratio was 1:5). All materials were adapted for this study in order to reflect the conflict between collective and individual interests. To reflect collective interests, the collective identity of the participants was emphasized in the materials, e.g., “You are the commander of a military base”; to reflect individual interests, the few that would be sacrificed were set to be family members (or include family members), e.g., “There are 10 military personnel on board the plane, including your younger brother who is also a member of the military”.

Each of the four moral dilemmas was written in two versions, i.e., (i) an action moral dilemma, in which the participant needed to decide whether to actively sacrifice individual interests to preserve the collective interests; and (ii) an omission moral dilemma, in which the participant was asked whether to prevent others from sacrificing the participant’s individual interests when they did so to preserve the collective interests. Each participant completed a total of eight moral decisions, and the order of the eight moral dilemmas was randomized.

The presentation of the moral dilemmas was the same as in Study 1. A moral dilemma (the cave dilemma) is listed below as an example:

You are the chief leader of an expedition. Your brother, who is also an explorer, is leading a group of five explorers, as the leader of the expedition team, into a cave located by the sea. When they intend to get out, your brother gets stuck at the exit of the cave, which leaves the other explorers stranded in the cave. The sea is about to rise, and the tide will soon flood the cave, and unless your brother stops blocking the cave entrance, all five explorers will drown, except for your brother (whose head is exposed).

**Action Moral Dilemma:** You discover the perilous situation of the expedition team and realize that there is nothing that can be done to save the five explorers unless the cave entrance is blown open with a bomb. Blowing open the hole will kill your brother, but if you don’t, all five explorers will drown. Would you use a bomb to blow up the hole?

**Omission Moral Dilemma:** You discover the perilous situation of the expedition team and realize that there is nothing that can be done to save the five explorers unless the cave entrance is blown open with a bomb. Blowing the hole will kill your brother, but without doing so, all five explorers will drown. The other members of the expedition are planning to use a bomb to blow up the hole, will you stop them from blowing up the hole?

**Dot Matrix Memory Cognitive Load Task:** The dot memory cognitive load task in Study 2 was the same as in Study 1.

### 3.2. Results

#### 3.2.1. Dot Matrix Memorization Load Task

The average correctness of the dot matrix memorization load task was 91.15% (*M* = 14.58, *SD* = 1.36). In the low-load condition, the mean correctness reached 88.88% (*M* = 14.22, *SD* = 1.36). In the high-load condition, the average correctness reached 92.38% (*M* = 14.94, *SD* = 1.29). This indicates that most of the participants performed well in the cognitive load task. However, there were still participants with low correct memorization rates, which implies that participants may have neglected the cognitive load task if they did not memorize the matrix correctly. To avoid this potential effect, data from participants with a correctness rate of 75% or less (*n* = 2, 2%) were excluded from this study (Bialek & De Neys, 2017). The final data consisted of 98 participants (76 females; mean age = 22.01, *SD* = 2.67), with a mean correctness rate of 89.81% (*M* = 14.37, *SD* = 1.14) for memorization in the low-load condition and 93.88% (*M* = 15.02, *SD* = 1.17) in the high-load condition.

#### 3.2.2. Moral Judgments

Participants’ collective-interest decisions were coded as “1”, and individual-interest decisions were coded as “0”.

A 2 (cognitive load: high load/low load) × 2 (action propensity: action/omission) two-way repeated-measures ANOVA was conducted on collective-interest decisions, and the results of the descriptive statistics are shown in Table 1.

The ANOVA results showed a significant main effect of cognitive load: *F*(1, 96) = 9.04, *p* = 0.003, *η_p_*^2^ = 0.09. Participants in the high-load group (*M* = 0.50, *SD* = 0.35) showed higher willingness to sacrifice the loved person for the sake of the collective good compared to the participants in the low-cognitive load group (*M* = 0.34, *SD* = 0.35). The main effect of preference for action was significant: *F*(1, 96) = 7.82, *p* = 0.006, *η_p_*^2^ = 0.08. Participants were more inclined to make choices consistent with the collective interest in omission moral dilemmas (*M* = 0.46, *SD* = 0.29) compared to action moral dilemmas (*M* = 0.38, *SD* = 0.31). The interaction between cognitive load and action propensity was not significant: *F*(1, 96) = 0.19, *p* = 0.662, *η_p_*^2^ = 0.00. Based on the effect size of the main effect of cognitive load, the statistical power was calculated using G*Power 3.1’s post hoc test. The results showed that 1 − *β* = 0.98.

#### 3.2.3. Identity

In this study, to highlight the conflict between collective and individual interests, the collective identity of the participants was emphasized in each of the materials (same railroad company employee/military base commander/same hospital security guard/expedition team chief). A 2 (cognitive load: high/low) × 2 (action tendency: action/omission) × 4 (identity: same railroad company employee/military base commander/same hospital security guard/expedition team chief) repeated-measures ANOVA indicated a significant main effect of cognitive load: *F*(1, 96) = 9.04, *p* = 0.003, *η_p_*^2^ = 0.09; a significant main effect of action propensity: *F*(1, 96) = 7.82, *p* = 0.006, *η_p_*^2^ = 0.08; and a significant main effect of identity: *F*(3, 96) = 6.16, *p* < 0.000, *η_p_*^2^ = 0.06 (see Table 2). The interaction between identity and cognitive load was significant: *F*(1, 96) = 6.16, *p* < 0.001, *η_p_*^2^ = 0.06, while other second- and third-order interactions were not significant (*p* > 0.05). Based on the effect size of the main effect of identity, the statistical power was calculated using G*Power’s post hoc test. The results showed that 1 − *β* = 1.00.

We used syntax in SPSS 26 to perform a simple test (the LSD test). When the identity was the railroad company’s employee, participants in the high-cognitive load group were more likely to choose collective interest than participants in the low-load group (*p* < 0.001). When the participants identified as security guards of the same hospital, participants in the high-cognitive load group were significantly more willing to sacrifice individual interests for the collective good compared to participants in the low-cognitive load group (*p* = 0.008). Nevertheless, there was no significant difference between participants in the high- and low-cognitive load groups in their tendency to sacrifice individual interests to preserve the collective interest when identifying as military base commanders (*p* = 0.499) and expedition chiefs (*p* = 0.988).

### 3.3. Discussion

This study indicates that cognitive load influences participants’ judgments in distinct ways during moral dilemmas, depending on the levels of activated collectivist and self-interested intuitions. When there is a significant disparity in the intensity of activated collectivist and self-interested intuitions, participants tend to initially decide in favor of the collective good, driven by stronger collectivist intuitions. This initial decision is less likely to be swayed by cognitive load. This finding aligns with our hypothesis and supports the hybrid dual-process model (Bago & De Neys, 2019a, 2019b).

Study 2 revealed that participants in the high-cognitive load group were more inclined to sacrifice individual interests for the collective good compared to those in the low-cognitive load group. This was observed in both action and omission moral dilemmas, aligning with our expectations. As praise judgments supplanted blame judgments and the action–omission effect disappeared (Bostyn & Roets, 2016), cognitive load ceased to differentially influence individuals’ decisions to “sacrifice one to save five” in both active and passive moral dilemmas. Cognitive load, therefore, appears to increase individuals’ reliance on praise judgments when making decisions that favor collective interests.

Regarding the impact of identities, no significant difference was observed in collective-interest judgments between the high- and low-cognitive load groups when the protagonist’s identity in moral dilemmas was a military base commander or an expedition leader. However, when the collective identity was a railroad company employee or a hospital security guard, participants in the high-cognitive load group made more decisions favoring collective interests compared to the low-cognitive load group. This suggests that the identities of the expedition leader and the military base commander, which inherently involve the responsibility to protect the lives of their members, trigger stronger collectivist intuitions and weaker self-interested intuitions among participants. Lin and Huang (2012) proposed that Confucianism and collectivism positively influence ethical leadership in Chinese management.

## 4. General Discussion

A series of studies have sought to investigate the hybrid model, with the majority employing cognitive load and time-pressure manipulations to explore the presence or absence of utilitarian intuitions in individuals (Bago & De Neys, 2017, 2019a, 2019b; Rosas & Aguilar-Pardo, 2019). The current research focused on the influence of cognitive load on individuals’ final judgments in moral dilemmas, with varying strengths of intuition activated, in order to validate the hybrid dual-process model. The findings revealed that the impact of cognitive load on moral judgments in utilitarian–deontological moral dilemmas was moderated by action tendencies. Facing action moral dilemmas, individuals under high cognitive load were more likely to make utilitarian choices compared to those under low load, while in inaction moral dilemmas, there was no difference in utilitarian choices between the high- and low-cognitive load groups.

In moral dilemmas involving collective–individual interests, cognitive load had an effect on moral judgments, and this effect was moderated by identity. The high-load group was more inclined to sacrifice individual interests for collective interests compared to the low-cognitive load group. Meanwhile, when the protagonists were identified as the military base commander and the expedition chief, there was no difference in collective-interest judgments between the high- and low-cognitive load groups. Conversely, cognitive load resulted in more collective-interest decisions in moral dilemmas where the collective identity was that of a railroad company employee or a hospital security guard.

Study 1 tested the validity of the hybrid model proposed by Bago and De Neys (2019a, 2019b) for explaining the mechanisms of moral judgments under cognitive load. Specifically, it examined whether the relative difference in the intensity of activation of utilitarian versus deontological intuitions influences the relationship between cognitive load and moral judgments. The findings contradictedstandard dual-process model (J. Greene & Haidt, 2002; J. Greene et al., 2004, 2008; J. Greene, 2015). According to Greene’s theory, individuals under high cognitive load should exhibit a decrease in utilitarian judgments due to their limited cognitive resources to activate utilitarian processing, that is, they are unable to revise their initial deontological responses. The results did not support this hypothesis, and the study did not find any influence of cognitive load on judgments, aligning with the results of previous studies on the relationship between cognitive load and moral judgments (e.g., Tinghög et al., 2016; J. Greene et al., 2008).

Given that classical moral dilemmas may conflate preferences for moral theories and action propensities (Crone & Laham, 2017; Gawronski et al., 2017), the current study considered action propensities (action/omission) as a variable. It was found that cognitive load did not significantly impact moral judgments in omission moral dilemmas. However, in action moral dilemmas, individuals made more utilitarian decisions under cognitive load.

The findings of this study suggest that the influence of cognitive load on moral judgments is moderated by the strength of the utilitarian pull in moral dilemmas. The hybrid model posits that initial responses can be either deontological or utilitarian, with subsequent revisions during deliberation depending on the relative activation strength of the two moral theories (Bago & De Neys, 2019a, 2019b). A smaller difference between the activation intensities of these two moral perspectives can increase the likelihood of decision alteration during deliberation. The action–omission effect on blame judgments implies that omission moral dilemmas exert a stronger utilitarian pull, as they absolve individuals from responsibility for outcomes, thereby activating stronger utilitarian intuitions than action moral dilemmas. Consequently, participants’ utilitarian decisions in these dilemmas were less influenced by cognitive load.

The increased utilitarian tendency under high cognitive load suggests that the process of moral judgments is not solely a path for utilitarian judgments to correct deontology. The simultaneous presence of utilitarian and deontological intuitions suggests that both can be revised during deliberation, aligning more closely with the assumptions of the hybrid dual-process model.

Greene’s dual-processing model associates deontological judgments with System 1 cognitive processing and utilitarian judgments with System 2 cognitive processing (J. Greene, 2015; J. Greene & Haidt, 2002). This model proposes that deliberation leads to a decrease in deontological judgments and an increase in utilitarian judgments. However, in recent years, several empirical studies have challenged this theory (e.g., Gamez-Djokic & Molden, 2016; McPhetres et al., 2018; Gurcay & Baron, 2016). A mouse-tracking study (2016) discovered that people may change their initial deontological choices to utilitarian choices, and they may also change their utilitarian choices to deontological choices. Tinghög et al. (2016) found that individuals are more likely to make utilitarian decisions in the footbridge moral dilemma under time pressure. The hybrid dual-processing model emphasizes that deontological intuition and utilitarian intuition both exist. Utilitarian intuitions may also be corrected under the action–omission effect, as individuals realize that they need to take responsibility for the outcome during the deliberation process, which reduces utilitarian judgments, and so show a lower tendency toward utilitarianism in the low-cognitive load condition (Bago & De Neys, 2019a, 2019b; Rosas & Aguilar-Pardo, 2019).

Also, the evidence at the level of single-celled organisms (Physarum polycephalum) shows that distinct behavioral patterns emerge under stress (lateral inhibition) versus safety (lateral activation). Stress induces more concentrated behavior, which in turn enhances the implementation of logic gates by these organisms through their motor responses to external stimuli. Thus, logic is more effectively implemented by Physarum polycephalum under stress conditions (Schumann, 2019; Schumann & Kuznetsov, 2018; Shirakawa et al., 2020). This is similar to the impact of cognitive load on moral judgments explored in the current research. Just as single-celled organisms tend to exhibit more concentrated behavior under stress, which leads to more effective logic, humans under high cognitive load may also rely more on intuition and make more utilitarian choices that weigh costs and benefits. This concentration of behavior under stress may provide a biological basis for understanding how cognitive load modulates moral judgments.

This reminds us that the role of cognitive processing in moral judgments is far more complex than the assumption of Greene’s standard dual-process model (J. Greene et al., 2004; J. Greene et al., 2008). Also, the factors that can influence moral judgments are not only different levels of utilitarian and deontological intuition, but also the action–omission effect of blame judgments. The interaction between cognitive load and identity, as identified in Study 2, further validates the rationality of the hybrid model and expands its applicability. Collective–individual-interest judgments were not affected by cognitive load when participants identified as military base commanders and expedition chiefs. However, the high-cognitive load group showed a greater willingness to sacrifice individual interests than the low-load group when the protagonists were employees of a railroad company and hospital security guards. These results align with the hybrid model because different protagonist identities represent different levels of concern for collective interests, and these influence the final moral choices. The military commander and the expedition chief, compared to the railroad company employees and the hospital security guards, carry more responsibility to protect the collective interest. When participants identify as these collective identities, the activated collectivist intuition will be significantly greater than the self-interested intuition, and their judgments will be less likely to be altered by cognitive load.

However, the interaction between cognitive load and action propensity on moral judgments was not significant in Study 2. In Study 1, due to the action–omission effect on blame judgments, inaction moral situations were more likely to activate participants’ utilitarian intuitions. In contrast, Study 2 shifted the focus of collectivist interest judgments from “harming others” to “consciously and willingly sacrificing one’s individual interests for the collective good”. Consequently, the individual’s action–omission effect was no longer automatically activated, rendering the interaction between cognitive load and action propensity insignificant.

It is interesting to note that this finding also seems to be explained by the standard dual-processing model. Participants’ were initially activated with collectivist intuitions. Then, individuals weigh the loss of personal interests when cognitive resources are sufficient, but when cognitive load depletes cognitive resources, individuals will be more inclined to sacrifice individual interests for the collective interests because they do not have enough resources to weigh their personal interests. This reminds us that the standard dual-processing model is not incorrect, just incomplete, as evidenced by the large body of research that still supports the standard dual-process model (e.g., Conway et al., 2018; Patil et al., 2021). The hybrid model is not a wholesale rejection of the standard dual-processing model, but rather complements and extends it.

Therefore, the hybrid model provides us with ideas to understand the different effects of cognitive load on ethical judgments in previous studies. The inconsistency of the results across studies may be because researchers used different moral dilemmas, ignoring the differences in the strength of activating utilitarian and deontological intuitions in various types of moral dilemmas. For example, J. Greene et al. (2008) used high-conflict personal dilemmas and impersonal dilemmas and did not find an effect of cognitive load on utilitarian tendencies, whereas Bialek and De Neys (2017) set up conflictual moral dilemmas (the cost of action was less than the benefit) and non-conflictual moral dilemmas (the cost of action was greater than the benefit), and the results showed no main effect of cognitive load, but there was an interaction effect of conflict. In conflict moral dilemmas, cognitive load led to lower utilitarian tendencies. Rosas et al. (2019) quantitatively manipulated the utilitarian pull in moral dilemmas, distinguishing between low-, medium-, and high-conflict-level moral dilemmas. Based on the hybrid dual-process model, cognitive load plays different roles in moral dilemmas.

Studies 1 and 2 not only illustrate that the contextual framing of moral dilemmas influences individual intuition, thereby affecting moral judgments, but also suggest that the intrinsic mechanisms of utilitarian–deontological and collective–individual-interest moral judgments may differ. Despite sharing a similar structure, the “kill one to save five” behavior assumes distinct roles in these two types of moral dilemmas. This highlights the importance of focusing on collectivist morality and broadening the scope of moral judgment studies to encompass a wider array of moral situations.

We also gained new insights into collectivist morality in Chinese culture. In Study 2, the increase in collective-interest judgments under the cognitive load condition implies that individuals are more likely to unthinkingly give way to personal interests in favor of collective interests when cognitive resources are scarce. This fully reflects the influence of China’s long cultural tradition of collectivism and also proves that individual moral intuitions not only come from our innate psychological structure but are also closely related to our growth in a particular culture (Rosas & Aguilar-Pardo, 2019).

By investigating the effects of cognitive load on moral judgments across different dilemmas, this study validates the hybrid model, demonstrating its robustness as a supplement to the standard dual-processing model and its capacity to explain moral judgments in diverse contexts. Simultaneously, this study delves into individuals’ moral judgments amidst the conflict between collective and individual interests, thereby enriching our understanding of moral judgments within the context of Chinese collectivist culture.

### 4.1. Action Dilemmas and Omission Dilemmas

Regarding the design for action and omission that we adopted, scholars in the field of morality have conducted some theoretical discussions. Gawronski et al. (2017) proposed the CNI model, in which researchers also considered the issue of confounding between action tendencies and deontological–utilitarian inclinations. They designed two types of dilemmas—a proscriptive norm prohibits action and a prescriptive norm prescribes action—which were similar to the action and omission scenarios we used in the current study. However, Baron and Goodwin (2020, 2021) pointed out that this approach has problems. First, they argued that the moral norm prohibiting the active killing of others is stronger than the norm requiring action to prevent others from actively killing. Therefore, when making such a switch, the two moral norms being compared differ significantly in strength in people’s minds. Prohibitive norms are inherently stronger than prescriptive norms, and thus the “action bias” exists within deontological norms. Second, they noted that when actions in prescriptive norm scenarios (omission dilemmas) are transformed into counteractions against others’ actions, the element of others is introduced. This may hurt the decision-maker’s feelings, leading to retaliation against the person taking the counteraction. It may also violate the scope of authority, thereby weakening the effectiveness of such authority in the future, as it would make those responsible no longer take their duties seriously. Moreover, they argued that the fact that someone else has already made a decision may lead the decision-maker to believe that the other person knows something we do not. Therefore, they pointed out that in prescriptive norm scenarios (omission dilemmas), choices that are thought to be based on norms may actually be based on consequences.

Gawronski et al. (2020) provided responses and explanations in relation to this criticism. They first acknowledged that allowing someone to kill (a prescriptive norm) is weaker in moral strength compared to actively taking action to kill others (a proscriptive norm). However, they refuted Baron and Goodwin (2020) by arguing that this asymmetry is not sufficient to prove that the classification comparison between action dilemmas and omission dilemmas is invalid. As long as the direct outcomes of a given action and inaction remain the same (e.g., whether someone is killed or allowed to die, the loss of life is identical), this asymmetry is irrelevant.

They also pointed out that the criticism by Baron and Goodwin (2020) is based on an implicit background assumption that experimental manipulations of moral norms have construct validity only when the behavioral effects are driven by conscious deliberation about the moral norms. However, this explanation actually conflates behavioral effects with explanatory psychological structures and also overlooks an important theory in the field of moral research: that judgments in line with moral norms are not necessarily the product of conscious deliberation about the norms (J. Greene et al., 2008; Haidt, 2001). For example, according to J. Greene et al.’s (2008) dual-process theory of moral judgment, judgments in line with moral norms are driven by emotional processes that do not involve conscious deliberation about the norms.

We agree with the points of Gawronski et al. (2020). The use of action dilemmas and omission dilemmas as classifications does not necessarily require the involvement of a cognitive deliberation process. As we analyzed in the introduction and as our data suggest, individuals in omission dilemmas may not engage in deliberation but rather make choices based on intuition. Also, cognitive load may limit the cognitive resources available for deliberation, but it does not affect moral choices in omission dilemmas.

Therefore, we believe that the classification of action dilemmas and omission dilemmas can effectively distinguish participants’ action tendencies along the dual dimensions of deontology and utilitarianism and allow for further analysis and discussion.

### 4.2. Limitations

In both Study 1 and Study 2, the comparison was limited to the high and low variations in the activation of individuals’ utilitarian (collectivist) intuitions within the context of action and omission moral dilemmas. The research conducted by Rosas et al. (2019) introduced a utilitarianism pull gradient to assess individuals’ judgments across a range of dilemmas. Future research could employ this model to pinpoint the factors that trigger participants’ moral intuitions and examine the extent to which participants’ utilitarian and deontological intuitions are stimulated by various situational influences.

Meanwhile, in exploring collectivist morality, this study only focuses on the situation where the conflict between collective and individual interests is irreconcilable, but the connotation of collectivist morality is not limited to this. In addition to the situation in which individual interests are completely sacrificed, there may also be situations in which the fulfillment of individual interests is delayed and cannot be fully satisfied. In future research, more types of collective–individual-interest moral dilemmas can be taken into consideration.

In the field of moral judgment research, the paradigms currently employed by researchers include not only the classic moral dilemmas but also the process dissociation (PD) paradigm (Conway & Gawronski, 2013) and the CNI model (Gawronski et al., 2017). Previous studies have found that the effects of cognitive load on moral thinking and judgment, as revealed by several paradigms of moral judgment research, are not entirely consistent (Conway & Gawronski, 2013; Gawronski et al., 2017; J. Greene et al., 2008; Bialek & De Neys, 2017) because they considered different kinds of scenarios. This study focuses solely on the impact of cognitive load on moral judgments within the context of classic moral dilemmas. Future research could explore the effects of cognitive load on choices in self- vs. collective-interest dilemmas based on the process dissociation procedure or the CNI model.

Additionally, previous research has shown that moral decision-making may be influenced by factors such as gender, age, education level, and culture (Graham et al., 2011; Zulfiqar, 2023). However, in this study, since all the participants were Chinese university students, we were unable to consider differences in age, education level, and cultural background. Therefore, we are uncertain whether the findings in the university student population can be extended to a broader population. Future research could consider expanding the range of participants to include individuals from different age groups, education levels, and cultural backgrounds to verify the validity of the findings. Also, in Study 2, the proportion of female participants was high (77%). It is uncertain whether the results were affected by gender. Future research should increase the proportion of male participants to further verify these findings.

## 5. Conclusions

This study revealed that the impact of cognitive load on moral judgments is moderated by the contextual setting of moral dilemmas. Specifically, when faced with a conflict between utilitarianism and deontology, individuals in high-cognitive load conditions are more likely to make utilitarian decisions in action dilemmas compared to those in low-cognitive load conditions. Conversely, in omission dilemmas, cognitive load does not influence utilitarian decisions.

When considering dilemmas involving conflicts between collective and individual interests, individuals under high cognitive load are more inclined to sacrifice individual interests for the collective good compared to those in a low-cognitive load state. Furthermore, when individuals occupy roles that demand a greater consideration of collective interests, such as military officers or expedition leaders, they prioritize collective interests, and cognitive load does not affect their moral choices. In contrast, when individuals’ professional roles entail less collective responsibility, cognitive load increases the tendency to favor collective interests.

## Figures and Tables

**Table 1:** Means and standard deviations of utilitarian judgments by experimental condition.

	Cognitive Load	*M*	*SD*	*n*
Action Dilemma	Low load	0.31	0.31	48
	High load	0.44	0.29	50
	Total	0.38	0.31	98
Omission Dilemma	Low load	0.38	0.31	48
	High load	0.55	0.25	50
	Total	0.46	0.29	98

**Table 2:** Means and standard deviations of judgments by experimental conditions.

	Cognitive Load	*M* ± *SD*	
		Action Dilemma	Omission Dilemma
Employee of the same railroad company	Low load	0.10 ± 0.31	0.25 ± 0.44
	High load	0.50 ± 0.51	0.56 ± 0.50
	Total	0.31 ± 0.46	0.41 ± 0.49
Military base commander	Low load	0.46 ± 0.50	0.00 ± 1.01
	High load	0.48 ± 0.51	0.16 ± 1.00
	Total	0.47 ± 0.50	0.54 ± 0.50
Same hospital security guard	Low load	0.17 ± 0.38	0.33 ± 0.48
	High load	0.42 ± 0.50	0.48 ± 0.51
	Total	0.30 ± 0.46	0.41 ± 0.49
Expedition chief	Low load	0.50 ± 0.51	0.44 ± 0.50
	High load	0.38 ± 0.49	0.56 ± 0.50
	Total	0.44 ± 0.50	0.50 ± 0.50

## Data Availability

The original contributions presented in this study are included in the article. Further inquiries can be directed to the corresponding author.
